# Manganese oxide phases and morphologies: A study on calcination temperature and atmospheric dependence

**DOI:** 10.3762/bjnano.6.6

**Published:** 2015-01-06

**Authors:** Matthias Augustin, Daniela Fenske, Ingo Bardenhagen, Anne Westphal, Martin Knipper, Thorsten Plaggenborg, Joanna Kolny-Olesiak, Jürgen Parisi

**Affiliations:** 1 Fraunhofer Institute for Manufacturing Technology and Advanced Materials, Wiener Str. 12, 28359 Bremen, Germany; 2 Department of Physics, Energy and Semiconductor Research Laboratory, Carl von Ossietzky University of Oldenburg, 26129 Oldenburg, Germany

**Keywords:** electrocatalytic activity, in situ X-ray diffraction, manganese glycolate, manganese oxide nanoparticles, mesoporous α-Mn_2_O_3_

## Abstract

Manganese oxides are one of the most important groups of materials in energy storage science. In order to fully leverage their application potential, precise control of their properties such as particle size, surface area and Mn*^x^*^+^ oxidation state is required. Here, Mn_3_O_4_ and Mn_5_O_8_ nanoparticles as well as mesoporous α-Mn_2_O_3_ particles were synthesized by calcination of Mn(II) glycolate nanoparticles obtained through an economical route based on a polyol synthesis. The preparation of the different manganese oxides via one route facilitates assigning actual structure–property relationships. The oxidation process related to the different MnO*_x_* species was observed by in situ X-ray diffraction (XRD) measurements showing time- and temperature-dependent phase transformations occurring during oxidation of the Mn(II) glycolate precursor to α-Mn_2_O_3_ via Mn_3_O_4_ and Mn_5_O_8_ in O_2_ atmosphere. Detailed structural and morphological investigations using transmission electron microscopy (TEM) and powder XRD revealed the dependence of the lattice constants and particle sizes of the MnO*_x_* species on the calcination temperature and the presence of an oxidizing or neutral atmosphere. Furthermore, to demonstrate the application potential of the synthesized MnO*_x_* species, we studied their catalytic activity for the oxygen reduction reaction in aprotic media. Linear sweep voltammetry revealed the best performance for the mesoporous α-Mn_2_O_3_ species.

## Introduction

Manganese oxides are a class of inexpensive compounds with a high potential for nanostructuring, which makes them attractive candidates for various applications, for example, as basis materials in supercapacitors and electrodes for Li-ion accumulators [1–3]. They exhibit high catalytic activity for different oxidation and reduction reactions due to the diversity in their Mn*^x^*^+^ cation oxidation states as well as morphological characteristics [4–5]. Many manganese oxide phases consist of tunnel structures built from MnO_6_ octahedra; these tunnels facilitate the access of reactants to the active reaction sites as well as the absorption of small molecules within the structure. The latter property is especially useful for application as molecular sieves and absorbents for the removal of toxic species from waste gases such as carbon monoxide and nitrogen oxide [6–8]. Additionally, manganese oxide structures exhibiting oxygen vacancies provide additional active sites for reduction and oxidation reaction intermediates, especially those involving oxygen. These properties are especially important for catalytic applications such as water oxidation [9–11] and the oxygen reduction and evolution reactions in metal/air battery systems [12–16]. Additionally, the advantages of manganese oxides can be enhanced by nanostructuring of the different species, which was recently shown by Zhang et al. [17]. In their report, better cyclability of Li-ion cells was obtained with anodes consisting of mesoporous Mn_2_O_3_ particles compared to Mn_2_O_3_ bulk powder electrodes [17].

Several nanoscale manganese oxide compounds can be prepared via calcination processes from suitable precursors [7,18–20]. Whereas many synthetic protocols yield manganese oxide species at the nanometer scale [21], for example, precipitation or the solvothermal route, these methods require long reaction times in the range of hours (up to 24 h) and subsequent drying processes of up to 2 days [22–27]. The synthesis via oxidation of manganese metal nanoparticles by gas condensation must be followed by annealing in O_2_-containing atmospheres to obtain different manganese oxide species [28]. An advantage of the calcination route, on the other hand, is the conservation of the morphology and size of the precursor during this process, which is of special interest when considering the use of nanoscale precursor particles. Further advantages include a relatively short synthesis time of about 1 to 5 h and the fact that a single precursor can be used to obtain several different products. Additionally, the calcination procedure is the only way to obtain pure phase Mn_5_O_8_ [28–33].

Here, we present the synthesis of nanocrystalline Mn(II) glycolate by a polyol process and demonstrate its suitability as a precursor in the synthesis of different manganese oxides. The polyol process is a well-known route for the synthesis of metal glycolates, usually yielding disc-shaped particles with diameters and thicknesses in the range of 1 to 3 µm and 100 to 250 nm, respectively [19,29,34–35]. By applying milder reaction conditions (i.e., decreasing the synthesis temperature and increasing the reaction time), we obtained homogeneous, rectangular Mn(II) glycolate nanocrystals with diameters less than 25 nm. The preparation of nanoscale precursor particles with uniform morphology is advantageous for the further synthesis of manganese oxides, because the control of the morphology and size of the particles is a major issue for their catalytic applications. The subsequent calcination process yielded Mn_3_O_4_ and Mn_5_O_8_ nanoparticles as well as mesoporous α-Mn_2_O_3_ particles with high surface areas of 300, 30 and 20 m^2^/g, respectively. The nanostructures of the obtained MnO*_x_* particles make them attractive candidates as highly active compounds in the field of catalysis and other applications in the field of energy storage. Furthermore, the synthesis presented in this study provides easy access to three different nanostructured MnO*_x_* species via one calcination process. This is advantageous for the investigation of the properties of the manganese oxides, as it rules out any synthesis-caused effects. The temperature- as well as the time-dependent phase transformation processes occurring during the oxidation of Mn(II) glycolate to Mn_3_O_4_, Mn_5_O_8_ and α-Mn_2_O_3_ were studied by in situ XRD measurements. A detailed study of the structural parameters of the manganese oxide products obtained after calcination in a temperature range from 320 to 550 °C in Ar and O_2_ atmosphere was performed using powder XRD.

## Results and Discussion

### Precursor synthesis

**Figure 1:**
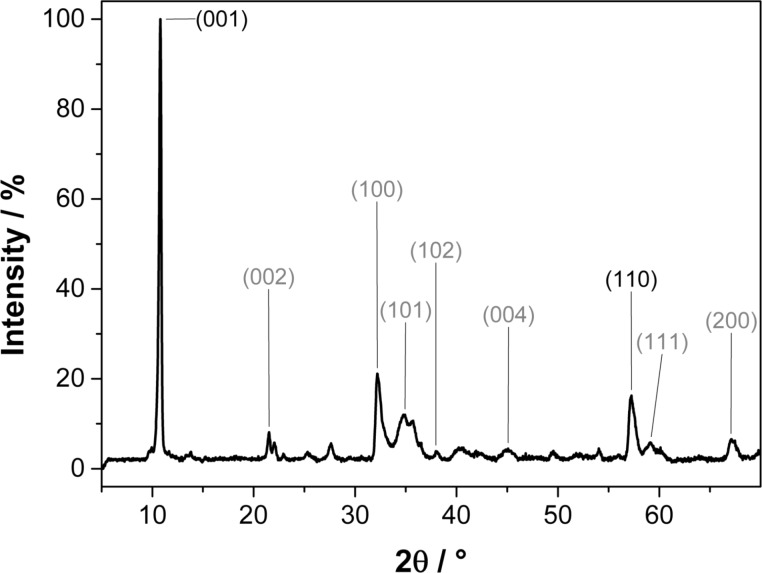
Powder XRD pattern of Mn(II) glycolate particles synthesized for 7 h; literature assignments [35] (black) and calculated reflection assignments (grey) are given for the brucite structure exhibiting lattice constants of *a* = *b* = 3.2 Å and *c* = 8.2 Å.

The polyol process reported by Liu et al. [19] was modified to yield the Mn(II) glycolate precursor for the thermal decomposition to the various manganese oxides. During the heating of the compound to 170 °C, a white precipitate appeared after 1 h, which was identified as manganese glycolate containing large impurities of the dehydrated educt Mn(II) acetate dihydrate and the product of a side reaction, manganese oxalate (MnC_2_O_4_, see Supporting Information File 1 for the powder XRD pattern of the product mixture). In order to obtain the pure Mn(II) glycolate precursor having homogeneous particle morphology, the reaction was continued at 170 °C until a white precipitate of pure Mn(II) glycolate appeared. This was verified by the X-ray diffraction pattern of the product after 7 h of synthesis as depicted in Figure 1. This product can be assigned to the trigonal brucite-type structure 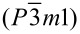 reported for Mn(II) glycolate by other groups [19,29,35]. A mean Scherrer crystallite size of 17 ± 8 nm was calculated for the Mn(II) glycolate particles. The interlayer distance along the [001] direction was calculated to be 8.2 Å. This value corresponds to the lattice constant, *c*, and is consistent with reports by other groups who measured lattice constants of *c* = 8.3 Å and *c* = 8.27 Å for Mn and Co glycolate, respectively [34–35]. As Sun et al. [35] did not use tetraethylene glycolate (TEG) in their synthesis, it is proposed here that TEG anions are not part of the Mn(II) glycolate structure presented in this report, as *c* would be increased even beyond 8.2 Å in this case. Hence, it can be concluded that TEG acts only as a stabilizing ligand to the Mn(II) glycolate particles. This and the milder synthesis conditions applied are considered to be the reasons for the relatively small crystallite sizes, differing by one order of magnitude from the data presented to date in the literature [19,29,35]. Inorganic compounds with a brucite structure such as Mg(OH)_2_, Co(OH)_2_, Ca(OH)_2_ and Ni(OH)_2_, exhibit lattice constant *c* between 4.6 and 4.9 Å and *a* in the range from 3.1 to 3.6 Å. For Mn(II) glycolate, the Mn–Mn distance (corresponding to lattice constant *a*) was calculated to be 3.2 Å from the (110) reflection, which is also in accordance with the findings of Sun et al. [35] who proposed that the structure widening in the *c* direction is due to the long-chain alcoholate anions interconnecting the metal–oxygen sheets in the *ab* plane of the unit cell.

**Figure 2:**
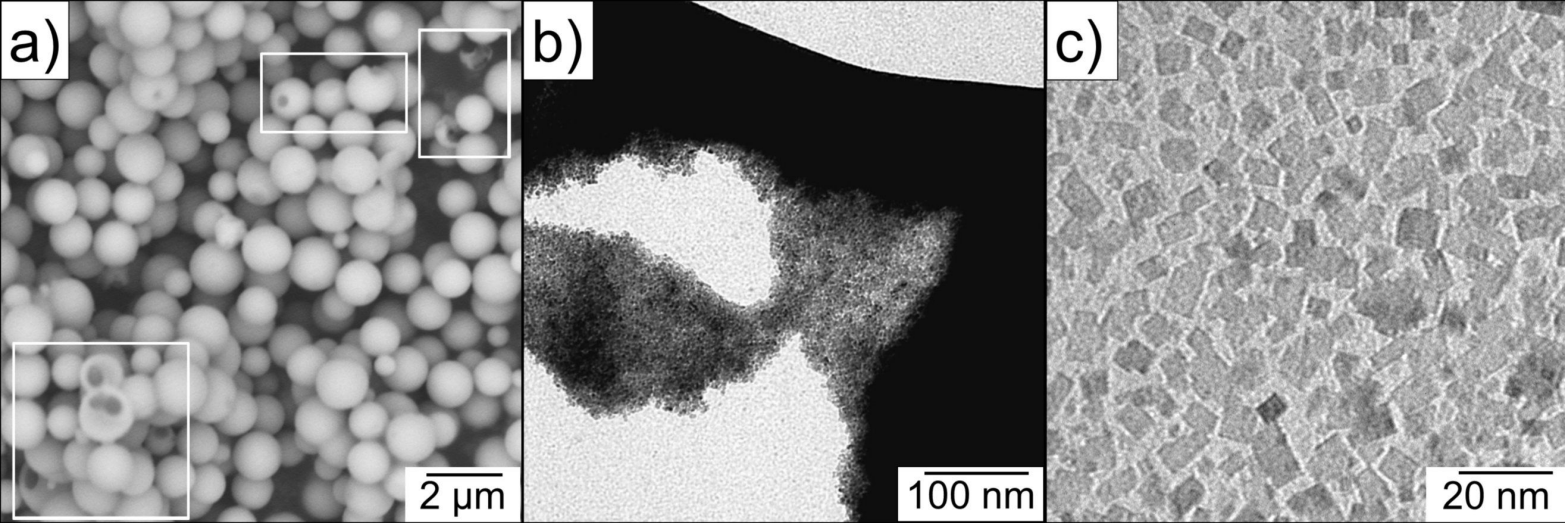
a) SEM and b) and c) TEM images of the Mn(II) glycolate particles. Particles with broken outer shells are highlighted by white frames in panel (a).

The morphology of the as-synthesized Mn(II) glycolate was investigated with SEM and TEM measurements. Figure 2a depicts SEM images of spherical Mn(II) glycolate particles with diameters up to 1 µm. These particles are hollow, which is deduced from the particles with broken outer shells (highlighted by white frames). Figure 2b shows a TEM image of one of these spherical particles, broken under the electron beam. A closer look reveals that the spheres are in fact agglomerates of rectangular Mn(II) glycolate nanoparticles with dimensions less than 15 nm (Figure 2c). The observed sizes of the particle are in good agreement with the calculated Scherrer crystallite sizes from the XRD pattern shown in Figure 1. The small dimensions of the particles make the synthesized Mn(II) glycolate a perfect precursor for the generation of manganese oxides by thermal decomposition processes.

### The oxidation process to different MnO*_x_* species

**Figure 3:**
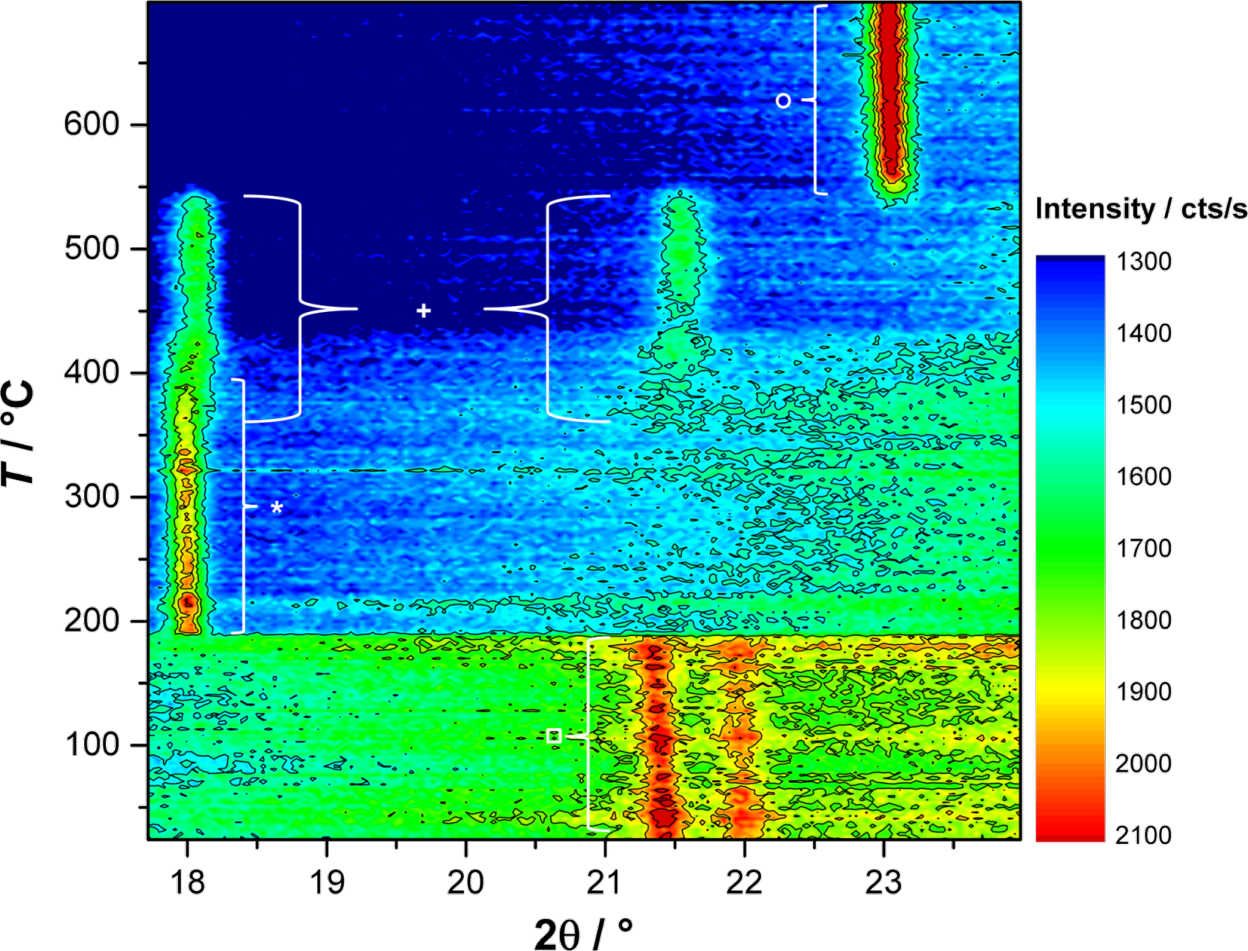
In situ XRD patterns recorded in a pure O_2_ flow while heating the Mn(II) glycolate precursor to 700 °C at 2 K/min; reflexes denoted are: Mn(II) glycolate (□), Mn_3_O_4_ (_*_), Mn_5_O_8_ (+) and α-Mn_2_O_3_ (○).

In order to investigate the temperature dependence of the oxidation process of Mn(II) glycolate, in situ X-ray diffractograms were recorded in the presence of O_2_ while heating the precursor to 700 °C at a heating rate of 2 K/min (see Figure 3). The 2θ region of 17.6–23.8° was monitored during the measurement, as it contains the reflections of the species that are most likely to be generated during the oxidation process (21.5° (Mn(II) glycolate, □) [19], 18.0° (Mn_3_O_4_, _*_) [36], 18.1° and 21.6° (Mn_5_O_8_, +) [31], and 23.2° (α-Mn_2_O_3_, ○) [37]). The reflection of Mn(II) glycolate at 21.5° is observed until a temperature of about 185 °C is reached, where a sudden decrease of the intensity (including the background intensity) is observed in the diffraction patterns due to the loss of organic species from the sample. This loss derives from the decomposition of the organic ligands and anions by oxidation; this is dependent on the temperature as well as on the partial pressure of oxygen. The Mn_3_O_4_ reflection at 18.0° immediately evolves at about 185 °C after the Mn(II) glycolate reflection has vanished. The appearance of the Mn_5_O_8_ reflection at 21.6° at about 350 °C is accompanied by the decreasing intensity of the Mn_3_O_4_ reflection at 18.0° as well as an increasing intensity of the Mn_5_O_8_ reflection at 18.1°, which is attributed to the slow oxidation of Mn_3_O_4_ to Mn_5_O_8_. The Mn_3_O_4_ reflection at 18.0° disappears at about 440 °C, indicating a completed oxidation process of Mn_3_O_4_ to Mn_5_O_8_. Both reflections assigned to Mn_5_O_8_ disappear at 550 °C after the appearance of the intense α-Mn_2_O_3_ reflection at 23.2° at a temperature of about 530 °C.

Hence, in O_2_ atmosphere, Mn_3_O_4_ is obtained at temperatures between 185 and 400 °C, Mn_5_O_8_ between 400 and 550 °C and α-Mn_2_O_3_ above 530 °C. This oxidation of Mn_3_O_4_ to Mn_5_O_8_ (rather than to Mn_2_O_3_) was found by Feitknecht [30] to take place during the heating of Mn_3_O_4_ particles at temperatures between 250 and 550 °C in an atmosphere containing more than 5% O_2_. Feitknecht attributed this Mn_3_O_4_/Mn_5_O_8_ phase transformation to a one-phase mechanism for Mn_3_O_4_ particles exhibiting BET surface areas of more than 10 m^2^/g. That is, the small particle diameters provide sufficient reaction sites for oxidation of the surface of the particles. Feitknecht also reported similar reflection intensities for Mn_3_O_4_ and Mn_5_O_8_ with a linear decrease and increase, respectively, during the oxidation process. The subsequent reduction process of Mn_5_O_8_ to α-Mn_2_O_3_ was observed by several groups to take place even in oxygen-containing atmospheres at temperatures greater than 500 °C [26,32,37].

**Figure 4:**
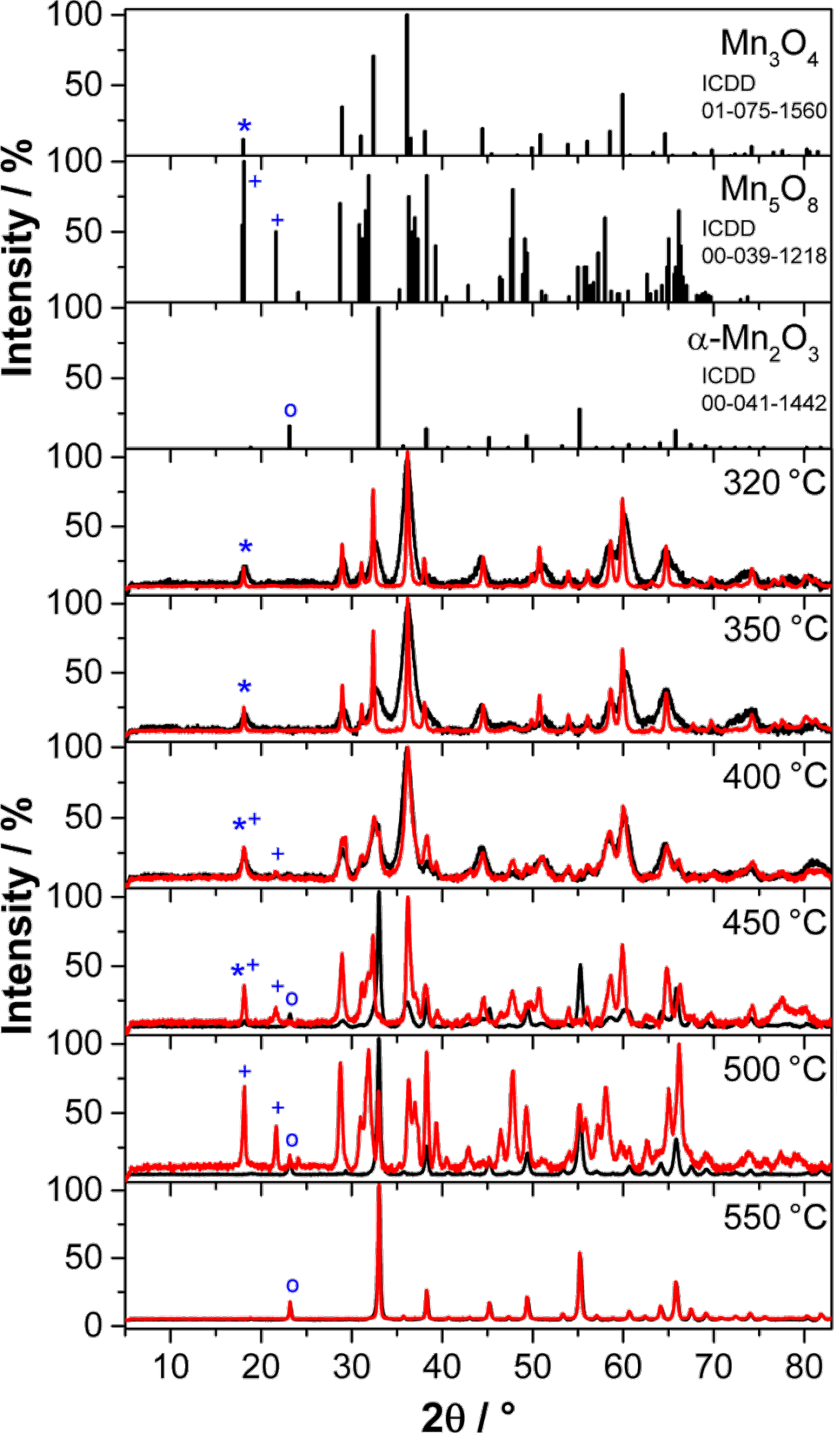
Powder XRD patterns of the manganese oxide particles obtained after calcination of the Mn(II) glycolate for 2 h at different temperatures in an Ar (black) and an O_2_ (red) flow of 50 NL/h. Reference patterns are depicted at the top for Mn_3_O_4_ (_*_), Mn_5_O_8_ (+) and α-Mn_2_O_3_ (○), where the symbols indicate the respective low-angle reflections.

The Mn(II) glycolate particles were calcined for 2 h in Ar and O_2_ atmospheres at different temperatures between 320 and 550 °C to investigate the dependence of the particle size, their morphology and the Mn*^x^*^+^ oxidation state in the resulting manganese oxide on the calcination temperature and atmosphere. The X-ray diffractograms of the resulting species as well as the reference patterns are shown in Figure 4; the crystalline phases observed in the XRD patterns are listed in Table 1.

**Table 1:** Crystalline manganese oxide phases obtained after calcination for 2 h in an Ar flow and an O_2_ flow (50 NL/h) at different temperatures; mean Scherrer crystallite sizes (from all assigned reflections) and lattice parameters (from the (101) and (004) reflections of Mn_3_O_4_ as well as the (400) reflection of α-Mn_2_O_3_) were calculated for samples yielding pure phases.

Temperature [ °C]	Atmosphere	Crystalline phase(s)	Lattice parameters [Å]	Crystallite size [nm]

320	Ar	Mn_3_O_4_	*a* = 5.72 *c* = 9.38	11 ± 3
	O_2_	Mn_3_O_4_	*a* = 5.75 *c* = 9.47	38 ± 11
350	Ar	Mn_3_O_4_	*a* = 5.69 *c* = 9.44	10 ± 3
	O_2_	Mn_3_O_4_	*a* = 5.74 *c* = 9.47	35 ± 10
400	Ar	Mn_3_O_4_	*a* = 5.73 *c* = 9.39	9 ± 2
	O_2_	Mn_3_O_4_, Mn_5_O_8_		
450	Ar	α-Mn_2_O_3_, Mn_3_O_4_		
	O_2_	Mn_3_O_4_, Mn_5_O_8_		
500	Ar	α-Mn_2_O_3_	*a* = 9.41	27 ± 4
	O_2_	Mn_5_O_8_, α-Mn_2_O_3_		
550	Ar	α-Mn_2_O_3_	*a* = 9.40	34 ± 5
	O_2_	α-Mn_2_O_3_	*a* = 9.41	44 ± 12

The tetragonal Mn_3_O_4_ phase (ICDD 01-075-1560, *I*4_1_/*amd*) is observed in the powder XRD patterns after calcination at temperatures between 320 and 450 °C in both atmospheres. It is, however, obtained as a pure phase only at temperatures up to 400 °C in Ar and up to 350 °C in O_2_ (see also Table 1). The presence of Mn_3_O_4_ in O_2_ atmosphere could also be observed in the in situ XRD measurements up to a temperature of 440 °C (see Figure 3). The lattice parameters and crystallite sizes of the pure Mn_3_O_4_ samples obtained by calcination in Ar as well as O_2_ atmospheres at 320 °C and 350 °C listed in Table 1 are obviously independent of the calcination temperature, but dependent on the calcination atmosphere. The samples obtained in Ar exhibit crystallite sizes of less than a third compared to those obtained in O_2_. Furthermore, the lattice constants of Mn_3_O_4_ produced in Ar are smaller at all temperatures than those obtained by calcination in O_2_ atmosphere. This could be due to oxygen vacancies, as the oxygen for the oxidation to Mn_3_O_4_ in pure Ar is only supplied by the manganese glycolate precursor and cannot be obtained from the gas atmosphere. The presence of oxygen vacancies is also supported by the less pronounced variation of the lattice constants of Mn_3_O_4_ obtained at 320 °C and 350 °C in O_2_ atmospheres, leading to the assumption of completely occupied oxygen sites in the structure of the oxide.

Cubic α-Mn_2_O_3_ (ICDD 00-041-1442, 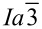) is obtained in Ar after calcination at temperatures between 450 and 550 °C and at 500–550 °C in O_2_. Pure-phase α-Mn_2_O_3_, however, is only obtained after calcination at temperatures of 500 and 550 °C in Ar and at 550 °C in O_2_ atmospheres (see also Table 1). The presence of α-Mn_2_O_3_ after calcination at 500 °C in O_2_ does not support the observations made in the in situ XRD measurements (compare to Figure 3), where a generation of α-Mn_2_O_3_ from Mn_5_O_8_ in an O_2_ atmosphere was only detected at temperatures above 530 °C. However, this is probably due to an additional time dependence of the phase transformation of Mn_5_O_8_ to α-Mn_2_O_3_, which was also suggested by Dimesso et al. [28]. In their report the α-Mn_2_O_3_ phase was observed to be the minor species second to Mn_5_O_8_ after calcination at 400 °C in air for 1 h, but was found to be the major species after calcination for 5 h at the same temperature. The lattice constants of the pure α-Mn_2_O_3_ phase obtained after calcination in Ar and O_2_, are obviously independent of the temperature and the calcination atmosphere (see Table 1). Therefore, in contrast to the observations made for Mn_3_O_4_, the absence of O_2_ in the calcination atmosphere does not lead to an increase in the concentration of oxygen vacancies in the α-Mn_2_O_3_ structure, which would be high enough to significantly change the lattice constants. The values of the Scherrer-derived crystallite sizes, however, suggest a temperature dependence of the obtained α-Mn_2_O_3_ particle size in Ar atmosphere, which was not the case for the Mn_3_O_4_ particles. The Scherrer-derived size of the crystallites of the pure phase α-Mn_2_O_3_ obtained in an O_2_ atmosphere at 550 °C is one third larger than that calculated for particles obtained in Ar at the same temperature. Hence, similar to the Mn_3_O_4_ phases described above, the presence of O_2_ in the calcination atmosphere yields larger crystallites of the same product.

No pure phase of monoclinic Mn_5_O_8_ (ICDD 00-039-1218, *C*2/*m*) could be obtained by calcination at temperatures between 320 and 550 °C for 2 h in both atmospheres. However, after calcination in O_2_ atmosphere, a small fraction of Mn_5_O_8_ can be detected in the products obtained at 400 and 450 °C, while it forms the majority of the product obtained after calcination at 500 °C. This is in good agreement with the in situ XRD patterns recorded in O_2_ atmosphere, where Mn_5_O_8_ was generated at about 350 °C and decomposed at 550 °C (see Figure 3).

In order to obtain pure-phase Mn_5_O_8_ particles, a longer calcination time of 5 h at 400 °C in an O_2_ atmosphere was chosen based on the time profile from the in situ XRD measurements (see Supporting Information File 1 for further details).

The properties of the Mn_5_O_8_ sample were investigated in more detail and compared to those of the Mn_3_O_4_ and α-Mn_2_O_3_ samples obtained by calcination for 2 h in Ar at 350 °C and at 550 °C, respectively. These Mn_3_O_4_ and α-Mn_2_O_3_ samples were chosen as they exhibit the most interesting properties for possible catalytic applications due to their small particle sizes.

**Figure 5:**
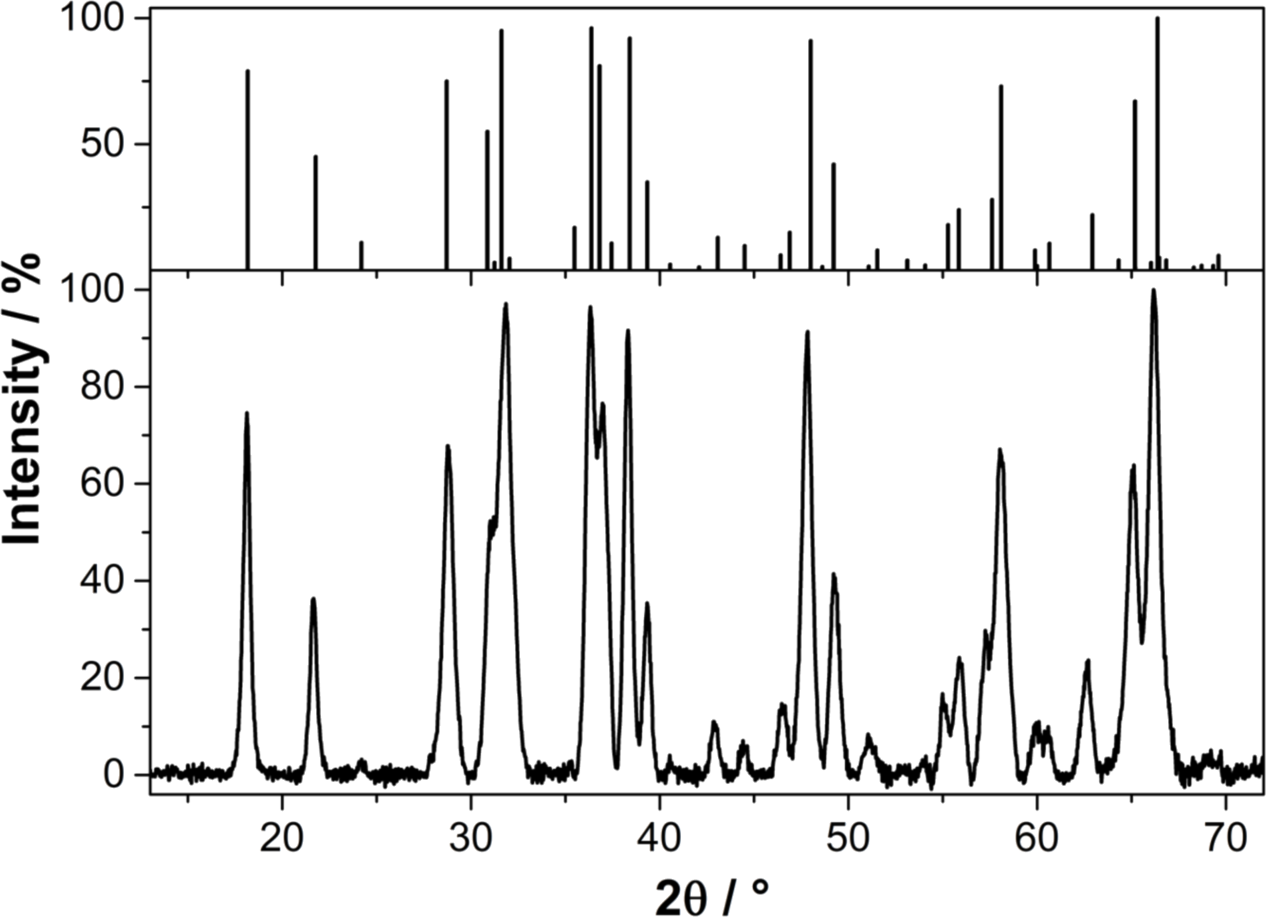
Powder XRD patterns of the Mn_5_O_8_ particles obtained by calcination of Mn(II) glycolate for 5 h at 400 °C in an O_2_ flow of 50 NL/h; a reference pattern is given in the top panel (ICDD 00-039-1218).

The X-ray diffraction pattern of pure Mn_5_O_8_ obtained by calcination in an O_2_ atmosphere at 400 °C for 5 h is shown in Figure 5. The Scherrer-derived crystallite size of this species is 22 ± 5 nm, and the lattice parameters of the monoclinic unit cell (ICDD 00-039-1218) are *a* = 10.40 Å, *b* = 5.73 Å and *c* = 4.87 Å with β = 109.6°, which is in good agreement with the data from literature (*a* = 10.34 Å, *b* = 5.72 Å and *c* = 4.85 Å with β = 109.25°) [31].

The temperature- and gas atmosphere-dependent oxidation process of Mn(II) glycolate to the manganese oxide species observed in the XRD patterns (see Figure 3 and Figure 4) was also investigated by thermogravimetric analysis (TGA). TGA measurements were recorded while heating the Mn(II) glycolate samples up to 700 °C with a heating rate of 2 K/min in an Ar (black) and an O_2_/Ar (1:2) (red) flow, respectively.

In both atmospheres a mass loss of 2.1% due to loss of water from the samples is detected up to a temperature of 150 °C. In Ar atmosphere, a further mass loss of 5.7% occurs up to 320 °C, which we attribute to the decomposition of the organic ligands (tetraethylene glycol and ethylene glycol), whose boiling points are in the temperature range of 150 °C to 320 °C. Subsequently, a mass loss of about 37% is detected up to 450 °C, which was also observed in TGA measurements of Ti(IV) glycolate by Jiang et al. [38] and was explained as a complete decomposition of the organic anions connecting the metal ions in that compound. Simultaneously, Mn(II) glycolate is oxidized to Mn_3_O_4_ and further to α-Mn_2_O_3_ between 185 °C to 450 °C, as was observed in the XRD measurements after calcination of the precursor for 2 h, as discussed above (see Figure 4). Although this oxidation process is accompanied by a decomposition of the organic groups, XPS results showed the presence of approximately 25 atom % carbon in the α-Mn_2_O_3_ species obtained after calcination for 2 h at 550 °C in Ar atmosphere. This, however, will not have a significant effect on its application as an electrocatalyst, as the reference and substrate material for the catalysts consists of carbon.

**Figure 6:**
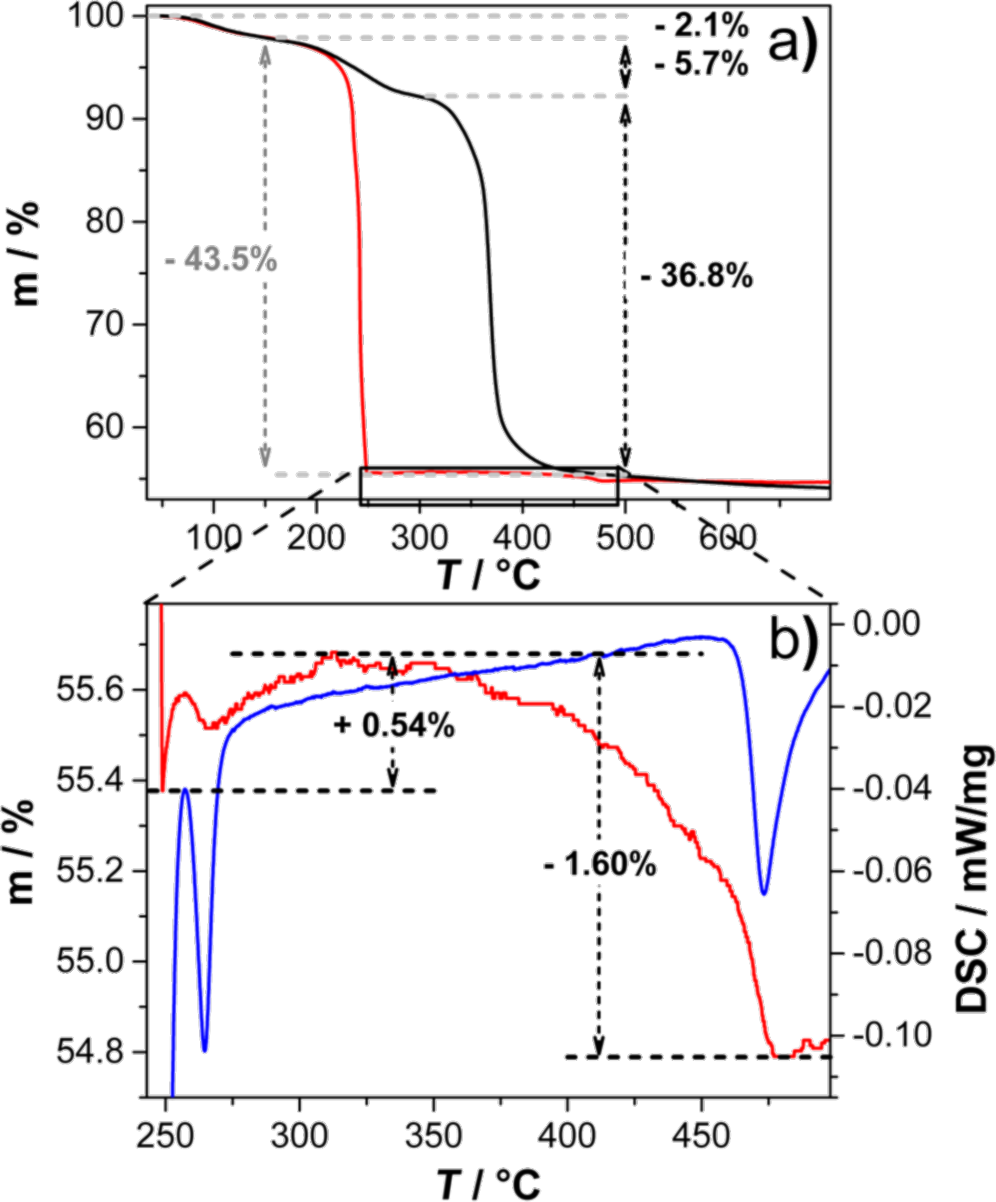
a) TGA measurements recorded while heating the Mn(II) glycolate precursor up to 700 °C at 2 K/min in an Ar (black) and an O_2_/Ar (1:2) (red) flow of 40 NmL/min; b) detailed view of the temperature range between 250 and 500 °C of the TGA (red) and DSC (blue) measurements in O_2_/Ar (1:2) flow indicating the oxidation of Mn_3_O_4_ to Mn_5_O_8_ and α-Mn_2_O_3_.

The decomposition of the organic species of Mn(II) glycolate in combination with an immediate oxidation to Mn_3_O_4_ in O_2_ atmosphere was observed at 185 °C in the in situ XRD measurement depicted in Figure 3. In the TGA measurement, however, a mass loss of 44% attributed to this process is detected between 150 and 250 °C. Hence, the observed mass loss includes the decomposition of organic species as well as the oxidation to Mn_3_O_4_. The temperature delay of the processes can be explained by considering the smaller O_2_ partial pressure of the atmosphere used for the TGA measurement. As both processes take place simultaneously, a clear assignment of the weight loss cannot be made. In order to investigate the processes subsequent to the large mass loss in the O_2_-containing atmosphere, a detailed view of the temperature region from 250 to 500 °C is shown in Figure 6b. Here, a mass increase of 0.54% is observed between 250 and 330 °C accompanied by a differential scanning calorimetry (DSC) signal of an exothermal phase transformation at 270 °C indicating a partial oxidation of Mn_3_O_4_ to Mn_5_O_8_. This is in good agreement with the development of the Mn_5_O_8_ phase observed at about 350 °C in the in situ XRD measurements (see Figure 3). The expected mass gain by complete oxidation to Mn_5_O_8_ of 5.59% is, however, much larger. Gillot et al. [27] proposed that heating rates between 1.2 and 2.5 K/min could lead to a direct oxidation of Mn_3_O_4_ to α-Mn_2_O_3_ even in O_2_ atmosphere, which would result in an expected mass gain of 0.97%. As both the direct oxidation of Mn_3_O_4_ to α-Mn_2_O_3_ and the oxidation via Mn_5_O_8_ would result in larger mass increases than the one observed in the measurement (0.54%), it is suggested that the decomposition of the organic species is not complete at a temperature of 330 °C. However, the subsequent mass loss of 1.60% from 330 to 480 °C, a DSC signal of an exothermal phase transformation at 480 °C, and the XRD measurements presented in this report (see Figure 3 and Figure 4) indicate the presence of Mn_5_O_8_. This mass loss again is lower than the expected value of 2.03% for a complete conversion of Mn_5_O_8_ to α-Mn_2_O_3_, which indicates that less α-Mn_2_O_3_ is formed from Mn_5_O_8_ than expected. Hence, in an O_2_/Ar atmosphere, α-Mn_2_O_3_ is generated partially from Mn_5_O_8_ and partially by direct oxidation from Mn_3_O_4_.

The increase and decrease in mass in the presence of gaseous O_2_ was proposed to be due to slow seed crystal oxidation of Mn_3_O_4_ to Mn_5_O_8_ for particles with BET surfaces larger than 10 m^2^/g and their subsequent transformation to α-Mn_2_O_3_ [29–30].

**Figure 7:**
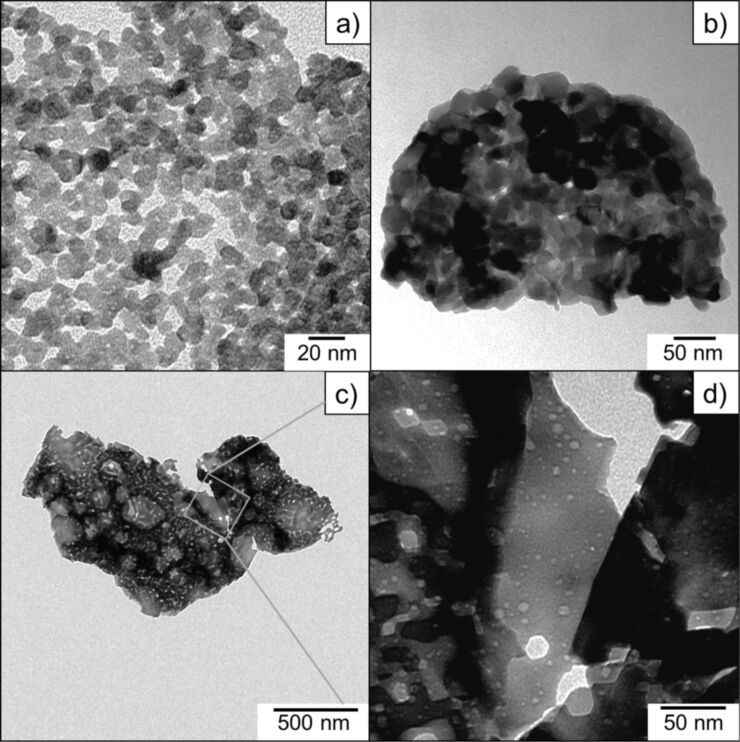
TEM images of the a) Mn_3_O_4_, b) Mn_5_O_8_, and c) and d) α-Mn_2_O_3_ samples.

**Figure 8:**
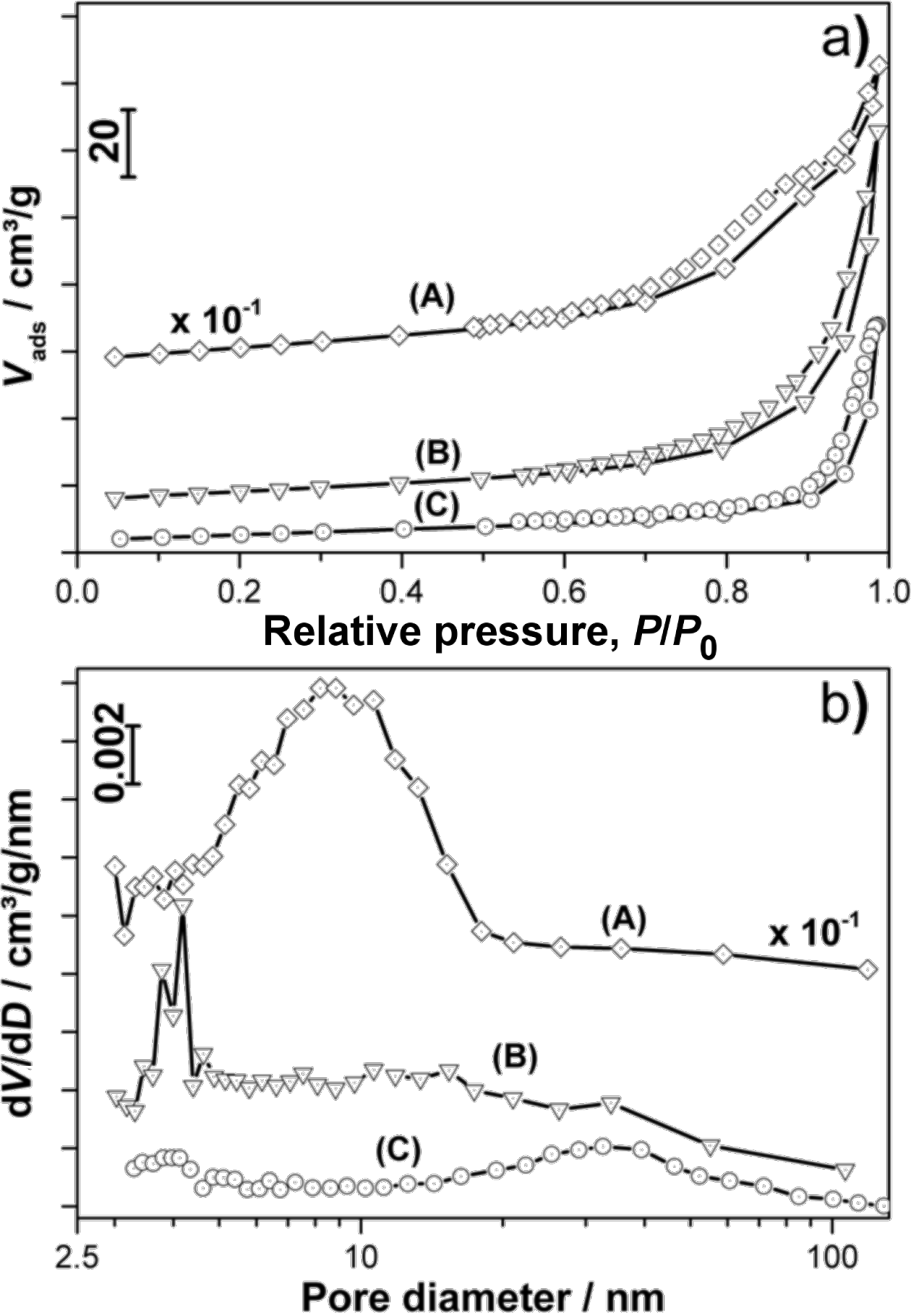
a) N_2_ adsorption–desorption isotherms (volume adsorbed versus relative pressure, *P*/*P*_0_) and b) the corresponding pore size distributions of the (A) Mn_3_O_4_, (B) Mn_5_O_8_ and (C) α-Mn_2_O_3_ samples.

In order to characterize the size of the particles and the active surface areas, pure Mn_3_O_4_, Mn_5_O_8_ and α-Mn_2_O_3_ species obtained by calcination of the Mn(II) glycolate precursor at temperatures of 350, 400 and 550 °C were characterized by TEM and BET measurements. The TEM images of the Mn_3_O_4_ and Mn_5_O_8_ samples are shown in Figure 7a,b; the sizes of the particles observed in TEM are in good agreement with the Scherrer-derived crystallite sizes calculated from the XRD patterns (see Figure 4 for comparison). The Mn_3_O_4_ sample shown in Figure 7a consists of a network of nanoparticles with diameters less than 10 nm and voids between the particles of about the same size. The same is true for the Mn_5_O_8_ sample in Figure 7b, but here the nanoparticles with diameters of up to 30 nm are obviously packed more densely. We attribute this to the increased temperature and duration of the calcination process, as well as to the presence of O_2_ in the atmosphere, which leads to larger particles, as previously discussed (see discussion for Figure 4). The α-Mn_2_O_3_ sample obtained by calcination in an Ar atmosphere (see Figure 7c,d), however, contains splinter-like pieces in the µm range (approximately 1–2 µm in the given TEM image) with a high concentration of holes with diameters of up to 20 nm, rather than individual nanoparticles. This manner of porosity for α-Mn_2_O_3_ was also confirmed by N_2_ adsorption–desorption measurements (see Figure 8). Mesoporosity in hexagonally shaped α-Mn_2_O_3_ particles and circular Mn_2_O_3_ discs obtained by calcination at temperatures above 600 °C was reported by several groups [17,37]. Ren et al. suggested that the mesopores are derived from a sequence of processes including Mn_5_O_8_ nanoparticle growth, rearrangement and merging during the transformation to α-Mn_2_O_3_ [37]. As the phase of Mn_5_O_8_ was not observed after calcination in Ar atmosphere, we propose that the presence of pores in the α-Mn_2_O_3_ particles reported here results from the analog growth, rearrangement and merging processes of the Mn_3_O_4_ nanoparticles.

The considerably smaller size of the crystallites obtained from the broadening of the XRD reflections of α-Mn_2_O_3_ is another argument for the porosity of these particles, as the pore walls in this case represent boundaries of the crystalline domains. Because these domains are regarded as Scherrer crystallites, we conclude that the obtained sizes are the mean distances between the pores as well as the minimum diameter of the α-Mn_2_O_3_ particles.

From the isotherms recorded during N_2_ adsorption–desorption measurements (see Figure 8a) specific BET surface areas of 302, 30 and 20 m^2^/g were calculated for the Mn_3_O_4_, Mn_5_O_8_ and α-Mn_2_O_3_ samples, respectively. The porosity of the α-Mn_2_O_3_ particles observed in the TEM images (see Figure 7c,d) is also supported by the N_2_ adsorption–desorption isotherms, which exhibit hysteresis. As hysteresis is also observed in the Mn_3_O_4_ and Mn_5_O_8_ nanoparticle isotherms (but not supported by observations made in the TEM images for these species, see Figure 7a,b), two different definitions of porosity can be applied for the different manganese oxide species. The pore size distributions depicted in Figure 8b show a pore diameter distribution between 3 and 20 nm with a mean pore diameter of 8.2 nm for the Mn_3_O_4_ sample. The porosity of the Mn_3_O_4_ nanoparticles can be explained by considering the voids between the particles in the network as the “pores” detected in the N_2_ adsorption–desorption measurements. This assumption is in good agreement with the similar sizes of the voids and nanoparticles observed in the TEM image (compared with Figure 7a). The same “pore definition” can be applied to the Mn_5_O_8_ sample exhibiting pore sizes between 3 and 5 nm with a comparably small mean pore size of 4.2 nm, probably due to the dense network of the particles observed in the TEM image (compared to Figure 7b). The α-Mn_2_O_3_ sample does not contain nanoparticles, but exhibits a pore size distribution between 3 and 5 nm as well as 10 and 90 nm with a mean diameter of 32.6 nm. In accordance with the TEM images (see Figure 7c,d for comparison) and the lowest BET surface area of all the samples, these pores do not derive from voids in the nanoparticle network but rather from the mesoporosity of the splinter-like pieces. A surface area of approximately 20 m^2^/g and pore diameters from 4 to 7 nm were also reported for Mn_2_O_3_ discs synthesized for the use as electrode material by Zhang et al. [17]. However, we believe a larger pore size to be advantageous for application as electrocatalysts, as electrochemical processes often produce solid products, which might easily clog pores in the micro- and meso-porous range.

The specific surface areas of Mn_3_O_4_ and Mn_5_O_8_ are in good agreement with the sizes of the particles observed in the TEM images and calculated from the XRD patterns, as smaller particles generally exhibit larger surface areas. However, the α-Mn_2_O_3_ particles, which are one order of magnitude larger, exhibit a high specific BET surface area comparable to that of the Mn_5_O_8_ nanoparticles. Two explanations for this large specific surface are proposed: the lower molar weight of α-Mn_2_O_3_ compared to Mn_5_O_8_ (resulting in a larger specific surface area), and the mesoporosity of the α-Mn_2_O_3_, which was already observed in the TEM images (see Figure 7c,d).

### Electrocatalytic activities of the MnO*_x_* species

In order to investigate the electrocatalytic activity of the synthesized MnO*_x_* species for the oxygen reduction reaction (ORR), linear sweep measurements were carried out.

**Figure 9:**
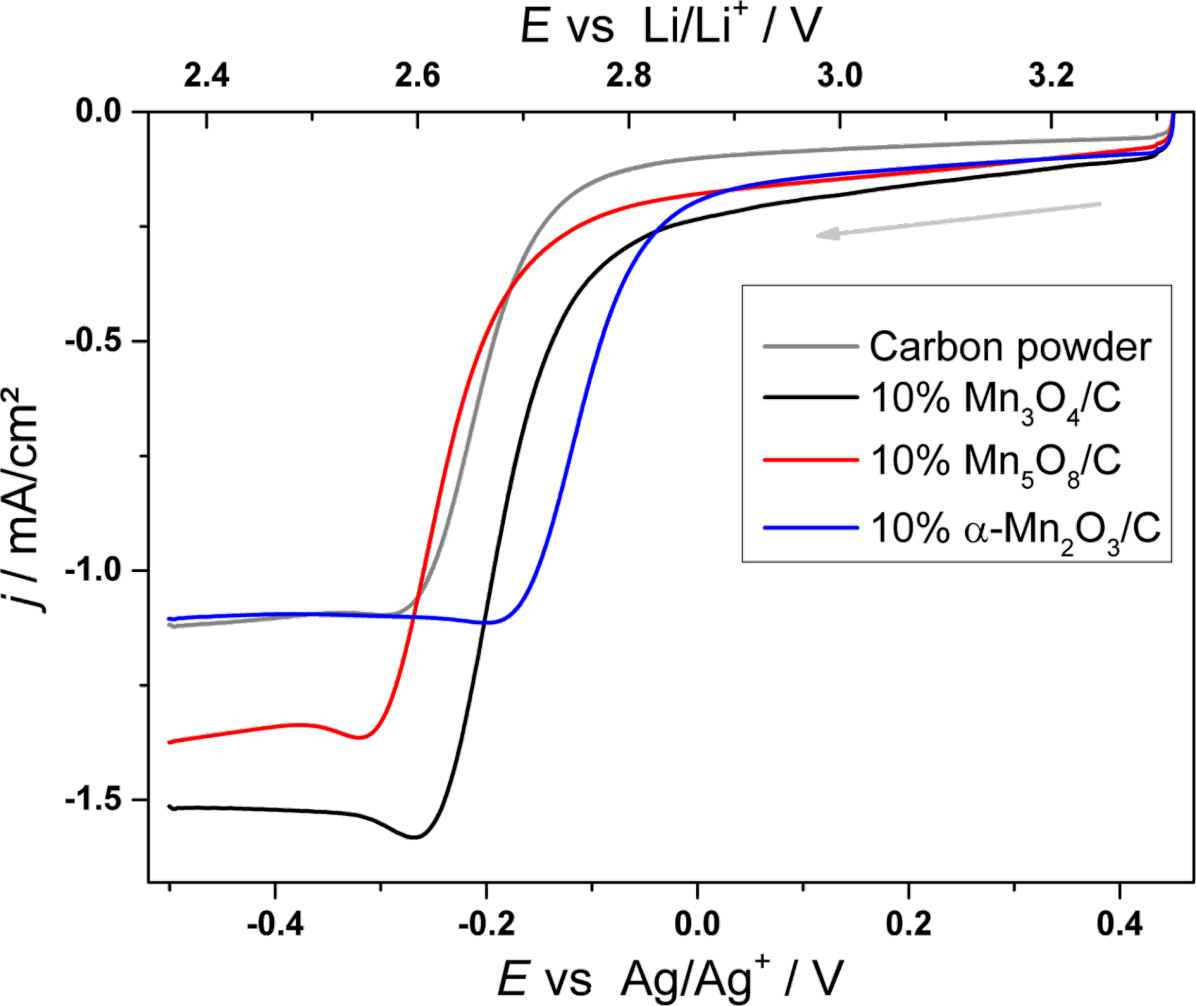
Linear sweep voltammograms of pure carbon powder (grey), Mn_3_O_4_/C (black), Mn_5_O_8_/C (red) and α-Mn_2_O_3_/C (blue) electrodes; electrolyte: 1 M LiTFSI/DMSO, cathodic scan direction, ν = 50 mV/s, ω = 200 rpm.

Figure 9 shows linear sweep measurements recorded at 50 mV/s comparing the activity of various 10% MnO*_x_*/carbon electrodes to a pure carbon electrode as a reference material for the ORR in aprotic electrolyte. The ORR peak potentials as well as the apparent reaction rate constant, 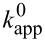, for the different electrode materials are summarized in Table 2. The reaction rate constant was calculated from:

[1]
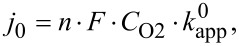


where *j*_0_ is the cathodic exchange current density (obtained from the Tafel plots of the linear sweep measurements), *n* = 1 is the number of transferred electrons, *F* is the Faraday constant, and *C*_O2_ = 2.1∙10^−6^ mol cm^−3^ is the oxygen solubility in DMSO [39].

**Table 2:** ORR potentials and reaction rate constants obtained from the linear sweep measurements recorded at ν = 50 mV/s.

	*E*_peak_ vs Li/Li^+^ [V]	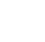 [10^−4^ cm/s]

Carbon (C)	2.58 ± 0.02	1.2 ± 1.1
Mn_3_O_4_/C	2.58 ± 0.08	2.6 ± 1.3
Mn_5_O_8_/C	2.58 ± 0.07	2.7 ± 2.3
α-Mn_2_O_3_/C	2.68 ± 0.05	4.5 ± 2.5

The mean ORR peak potential of the carbon reference material given in Table 2 is observed at 2.58 V. The only MnO*_x_* species with a significant increase of the ORR potential of 100 mV with respect to the carbon as well as the other MnO*_x_*/C electrodes is the mesoporous α-Mn_2_O_3_ catalyst. The obvious activity is reflected in the approximately four- and two-fold larger apparent ORR rate constant 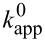 compared to the carbon and the other MnO*_x_*/C electrodes, respectively.

A detailed kinetic and mechanistic study on the electrocatalytic activities of the different MnO*_x_* species for the aprotic oxygen reduction reaction is reported elsewhere [40].

## Conclusion

In summary, a polyol synthesis was presented yielding rectangular, Mn(II) glycolate nanoparticles with dimensions of 17 ± 8 nm. Particle sizes of less than 100 nm are reported here for the first time. We attribute this small size of the particles to the stabilizing tetraethylene glycol ligand used during the synthesis, as well as milder reaction conditions compared with other reports (i.e., a decreased temperature and a longer reaction time). In situ XRD measurements showed the sequence of time- and temperature-dependent phase transformations during oxidation of the Mn(II) glycolate precursor to α-Mn_2_O_3_ via Mn_3_O_4_ and Mn_5_O_8_ in O_2_ atmosphere. Structural and morphological investigations revealed the dependence of the lattice constants and particle sizes of the MnO*_x_* species on the calcination temperatures in a range from 320 to 550 °C as well as on Ar and O_2_ atmospheres. Based on the results of these measurements, several manganese oxide species were synthesized by calcination of Mn(II) glycolate particles in argon and oxygen atmosphere at different temperatures. The calcination process yielded Mn_3_O_4_ nanoparticles with dimensions of about 10 nm and a surface area of 302 m^2^/g, Mn_5_O_8_ nanoparticles with diameters of 22 nm and a surface area of 30 m^2^/g as well as mesoporous α-Mn_2_O_3_ particles with mean pore diameters of about 33 nm and a surface area of 20 m^2^/g. The small dimensions of the particles and large surface areas of the manganese oxides presented here result from use of nanostructured precursor particles. Linear sweep measurements showed the activity of the mesoporous α-Mn_2_O_3_ species for the oxygen reduction reaction in aprotic media with respect to the observed potentials as well as an enhanced kinetic activity. The catalytic activity of different manganese oxides can be enhanced by a larger surface area, resulting from small particle dimensions or mesoporosity. This makes our synthesis a suitable process to obtain manganese oxides having properties of particular interest for electrochemical and chemical catalysis. Furthermore, the synthesis of manganese oxides via one route reported here is of additional interest, as it excludes any synthesis-caused effects on the products and allows investigation on the catalytic effect of similarly synthesized manganese oxides with different properties.

## Experimental

### Materials

Manganese(II) acetate tetrahydrate (MnAc_2_, >99%, pure) and ethylene glycol (EG, >99.5%, p.a.) were purchased from Carl-Roth. Tetraethylene glycol (TEG, 99%) was delivered by Sigma-Aldrich. For the electrode preparation, a 10 wt % Nafion^®^/water solution was purchased from Sigma-Aldrich, analytical reagent-grade ethanol from Fisher Scientific, and Vulcan^®^ XC72R carbon powder was obtained from Cabot. For electrochemical measurements, reagent-grade lithium bis(trifluoromethylsulfonyl)imide (LiTFSI) was purchased from Merck KGaA and dimethyl sulfoxide (DMSO, anhydrous, ≥99.9%) from Sigma-Aldrich. All chemicals were used without further purification.

### Synthesis of Mn(II) glycolate

In a typical reaction, 1 mmol (0.246 g) MnAc_2_ was mixed with 3 mmol (3 mL) TEG and added to 30 mL EG in a three-neck round-bottom flask. The solution was heated to 170 °C while stirring. Upon heating, the solution turned brown at a temperature of about 110 °C, and after further heating for about 1 h at 170 °C, a white precipitate appeared that disappeared again after 1 h. The solution was stirred for another 4 h at 170 °C until a white precipitate appeared again which indicated the formation of the Mn(II) glycolate particles. The product was stirred for another hour at 170 °C to complete the reaction and was subsequently cooled down to room temperature. The white powder was centrifuged and washed at least five times with ethanol to remove any impurities. Subsequently, the white product was dried under Ar flow.

### Synthesis of Mn_3_O_4_, Mn_5_O_8_ and α-Mn_2_O_3_

The obtained Mn(II) glycolate powder was calcined in an Ar flow of 50 NL/h for 2 h at 350 °C and at 550 °C yielding Mn_3_O_4_ and α-Mn_2_O_3_, respectively. Mn_5_O_8_ was obtained by calcination of the precursor in an O_2_ flow of 50 NL/h for 5 h at 400 °C.

### Characterization methods

Transmission electron microscopy (TEM) was carried out with a Zeiss EM 902A microscope with an acceleration voltage of 80 kV. For high resolution TEM (HR-TEM) measurements a JEOL JEM2100F microscope with an acceleration voltage of 200 kV was used. The samples for TEM and HR-TEM measurements were prepared by depositing a drop of an ethanol emulsion of the powder on a carbon-coated copper grid and drying at room temperature.

Scanning electron microscopy (SEM) was carried out with an Oxford INCA system employing a PentaFET Precision INCA X-act detector integrated into the Hitchai S-3200N microscope. The sample was prepared by depositing an ethanol emulsion of the sample onto an Al substrate and drying at room temperature.

For X-ray diffraction (XRD), a PANalytical X’Pert Pro MPD diffractometer was used operating with Cu Kα radiation, Bragg-Brentano θ-2θ geometry and a goniometer radius of 240 mm. Samples for XRD measurements were prepared by placing the powder onto low-background silicon sample holders. Different atmospheres were used as mentioned in the text. The crystallite sizes of the samples were calculated from all assigned reflections via the Scherrer equation. The lattice parameters were obtained with the Bragg equation from assigned diffraction reflections.

In situ XRD measurements were performed in the same geometry using a high temperature chamber from Anton Paar (HTK 1200N). The temperature profile measurement was recorded while heating the powder sample from 25 to 700 °C with a heating rate of 2 K/min. The time profile measurement (shown in Supporting Information File 1) was conducted by heating the powder sample from 25 to 400 °C with a heating rate of 18 K/min and subsequent constant heating at 400 °C for 350 min. The powder sample was placed on a corundum sample holder. During the measurement the thermal expansion was corrected automatically. The measurements were performed in an O_2_ flow.

Thermogravimetric analysis (TGA) and differential scanning calorimetry (DSC) were carried out with a Netzsch STA 449 F3 Jupiter thermo-analysis system. The sample was deposited in an Al_2_O_3_ crucible and heated from 35 to 700 °C with a heating rate of 2 K/min in an O_2_(6.0)/Ar(5.0) (1:2) and an Ar(5.0) gas flow of 40 NmL/min.

The porosity of the manganese oxides was determined by N_2_ adsorption–desorption measurements. Prior to the measurement, the material was kept for 18 h at 180 °C under vacuum to remove any residual gas and moisture from the sample. The adsorption–desorption isotherms were measured employing a Quantachrome Nova 2000E device at 77 K. The Brunauer–Emmet–Teller (BET) method was used to determine the complete inner surfaces *S*_0_ and the Barrett–Joyner–Halenda (BJH) method for mesopore surface analysis as well as the determination of pore size distributions.

### Electrode preparation

The catalyst/carbon ink for the powder electrodes was prepared by mixing and grinding 90 mg Vulcan^®^ XC72R carbon powder with 10 mg of MnO*_x_* catalyst. This active material was dispersed in ethanol and ultrasonicated for 20 min. As a binder material, 0.1 wt % Nafion/water solution was added to the catalyst/carbon paste and ultrasonicated for another 20 min. A 10 µL drop of the ink was applied on a glassy carbon disc (*d* = 0.5 cm) and dried for 12 h at 80 °C. The 10 wt % catalyst loading of the prepared electrodes equals 4.8 µg per electrode or 24.4 µg cm^−2^.

### Electrochemical measurements

For linear sweep voltammetry measurements, a Gamry Instruments Reference 600 Potentiostat was used. The measurements were carried out on a rotating disc electrode (RDE) in a glove box in Ar atmosphere at ambient temperature. For the electrochemical setup, glassy carbon (Pine Research Instrumentation, electrode model no. AFE3T050GC) and carbon/catalyst-coated glassy carbon discs (see above) served as working electrodes. A polished Ag wire and a Pt disc were used as reference and counter electrodes, respectively. 1 M LiTFSI/DMSO was used as the electrolyte, which was saturated with pure O_2_ for 25 min before the start of the measurement. The linear sweep measurements were recorded at a scan rate of ν = 50 mV/s and a rotational frequency of ω = 200 rpm.

## Supporting Information

The supporting information features the powder XRD pattern of Mn(II) glycolate particles after 1 h of synthesis at 170 °C in addition to in situ XRD patterns of the time-dependent oxidation of Mn_3_O_4_ to Mn_5_O_8_ at 400 °C in O_2_.

File 1 Additional XRD experimental data.
