# Supplementary material for: Manganese oxide phases and morphologies: A study on calcination temperature and atmospheric dependence
Source: Beilstein J Nanotechnol. 2015 Jan 6;6:47–59. doi: 10.3762/bjnano.6.6 (PMC4311619; doi:10.3762/bjnano.6.6)
Supplement: File 1 — Additional XRD experimental data. [file Beilstein_J_Nanotechnol-06-47-s001.pdf]

Supporting Information

for

Manganese oxide phases and morphologies: A study on calcination temperature and atmospheric dependence

Matthias Augustin*[1,2], Daniela Fenske[1], Ingo Bardenhagen[1], Anne Westphal[1], Martin Knipper[2], Thorsten Plaggenborg[2], Joanna Kolny-Olesiak[2], and Jürgen Parisi[2]

Address: [1]Fraunhofer Institute for Manufacturing Technology and Advanced Materials, Wiener Str. 12, 28359 Bremen, Germany and [2]Department of Physics, Energy and Semiconductor Research Laboratory, Carl von Ossietzky University of Oldenburg, 26129 Oldenburg, Germany

Email: Matthias Augustin* - matthias.augustin@ifam.fraunhofer.de

* Corresponding author

Additional XRD experimental data.

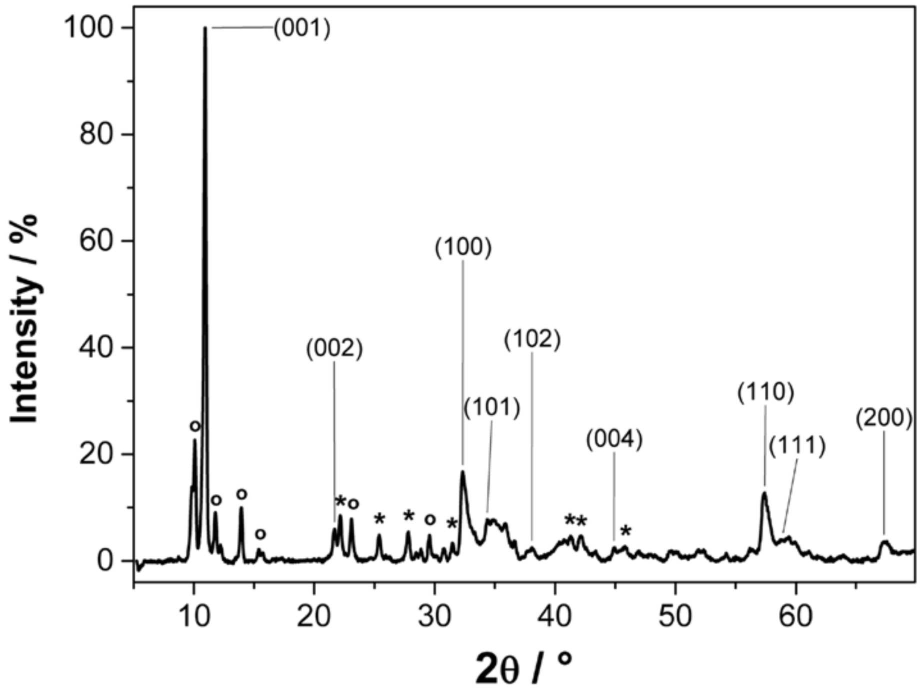

Figure S1: Powder XRD pattern of Mn(II) glycolate particles after 1 h of synthesis at 170°C; labels denote reflections assigned to manganese glycolate, circles and asterisks denote reflections of manganese acetate dihydrate (ICDD 00-056-0183) and the side product manganese oxalate (ICDD 00-032-0646), respectively.

The in situ XRD patterns depicted in Figure S2 were recorded to investigate the time-dependent oxidation of Mn_3O_4 (reflection at 18.0°, *) [36] to Mn_5O_8 (reflections at 18.1° and 21.6°, +) [31]. Therefore, after initially heating Mn(II) glycolate up to 400°C in an O_2 flow at 18 K/min the temperature was kept constant for 350 min. The reflection at 18.0° and the absence of a reflection at 21.5° indicate an already completed oxidation of Mn(II) glycolate to Mn_3O_4 at the start of the measurement. The Mn_5O_8 reflection at 21.6° appears after about 40 min of the measurement. The

slow oxidation process of Mn_3O_4 to Mn_5O_8 can also be observed by the decreased intensity as well as a broadening of the reflection at 18.0° up to 18.1° due to an overlap of the Mn_3O_4 and Mn_5O_8 reflections until about 150 min of the measurement, when the reflection at 18.0° disappears. For the subsequent 200 min of the measurement, the reflection is detected constantly at 18.1° indicating the end of the oxidation process, which was also observed in the temperature-dependent in situ XRD measurements as a slow process from about 350°C to about 440°C (compare with Figure 3). Although the background reflections of the in situ XRD patterns depicted in Figures 3 and S2 increase with increasing 2θ, the strong α-Mn_2O_3 reflection at 23.2° is observed in the temperature-dependent in situ XRD measurements (see Figure 3) but not in the time-dependent measurements at 400°C. This result is contrary to that reported by Dimesso et al. [28], who observed the formation of α-Mn_2O_3 during thermal treatment at 400°C in the presence of O_2 for more than 5 h. Our results show that by calcination at 400°C in an O_2 flow for a time between 150 and 300 min, Mn_5O_8 is at least the major, if not the only species present, without any indication of the formation of α-Mn_2O_3. After heating at 400 °C for more than 300 min in O_2 atmosphere, Mn_5O_8 is the only manganese oxide phase in the product, which was shown by X-ray diffraction after calcination (compare to Figure 5).

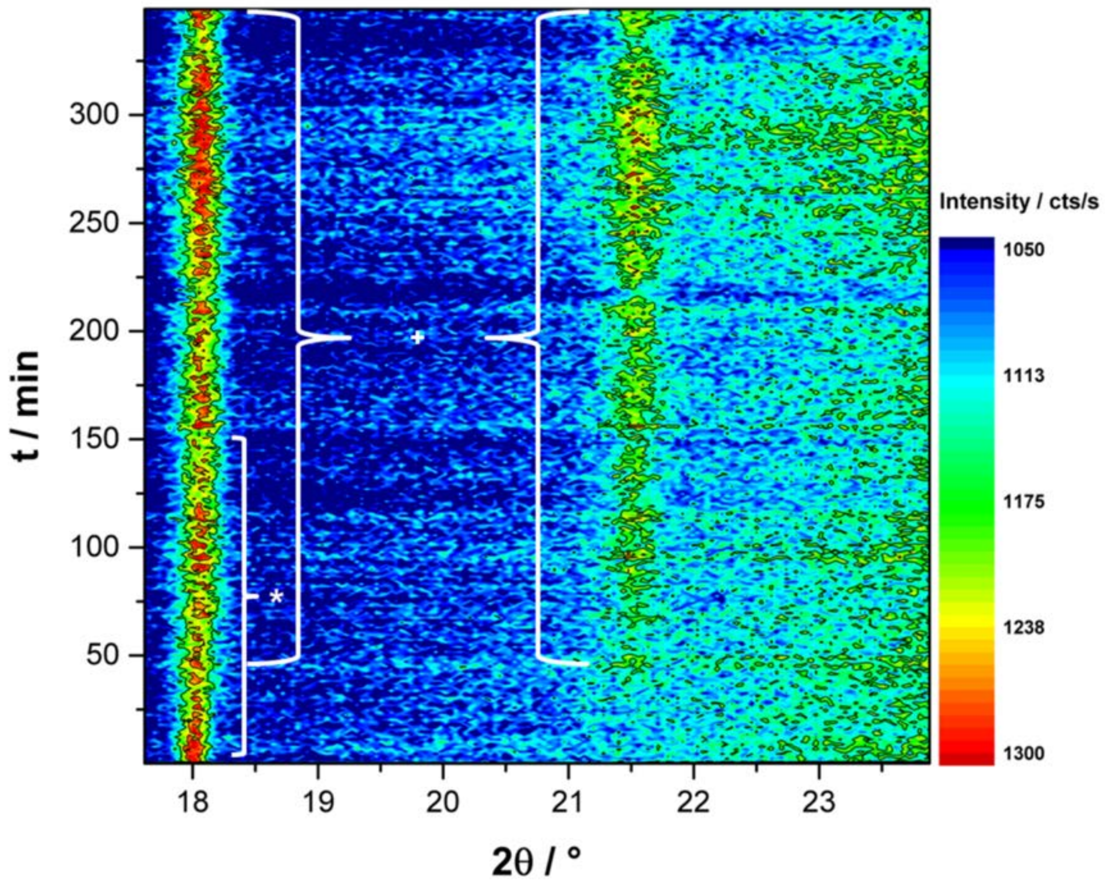

Figure S2: In situ XRD patterns of the Mn(II) glycolate particles heated for 350 min at 400°C in a pure O_2 flow; reflexes denoted are: Mn_3O_4 (*) and Mn_5O_8 (+).
